# Prevalence and perception of pre-morbid lifestyle-related risk factors among covid-19 survivors in Lagos state and Abuja capital city of Nigeria

**DOI:** 10.1186/s12889-024-19502-w

**Published:** 2024-07-17

**Authors:** Ifeoma N Monye, Tijani Idris Ahmad Oseni, Moyosore T. Makinde, Abiodun B. Adelowo, Safiya Yahaya-Kongoila, Marvellous C. Njoku-Adeleke, Aramide Oteju, Samba Nyirenda, Temitayo O. Elebiyo, Ijeoma Judith Dozie, Chinasa T. Ugwuegbulem-Amadi

**Affiliations:** 1 Society of Lifestyle Medicine of Nigeria (SOLONg), Lagos, Nigeria; 2 Brookfield Clinics Centre for Lifestyle Medicine, Department of Family Medicine, National Hospital, Abuja, Nigeria; 3 Department of Family Medicine, Edo State University, Edo State University Teaching Hospital, Uzairue, Auchi, Edo State Nigeria; 4 Department of Family Medicine, Lagos State University Teaching Hospital, Ikeja, Lagos Nigeria; 5 Niger Delta Power Holding Company, Abuja, Nigeria; 6 Department of Paediatrics, Wuse District Hospital, Abuja, Nigeria; 7 Department of Family Medicine, Lagos University Teaching Hospital, Lagos, Nigeria; 8 Sarai Holistic Care, Francistown, Botswana; 9 Department of Family Medicine, University of Benin Teaching Hospital, Benin City, Nigeria; 10 Department of Family Medicine, Federal University Teaching Hospital, Owerri, Imo State Nigeria; 11 Ariella Health and Fitness Limited/Queen of Peace Hospital, Port Harcourt, Rivers State Nigeria

**Keywords:** Covid-19 survivors, Lifestyle, Nigeria, Perception, Prevalence, Pre-morbidity

## Abstract

**Introduction:**

This study investigated the prevalence and perception of premorbid lifestyle-related risk factors among Covid-19 Survivors in Abuja and Lagos, Nigeria.

**Methodology:**

A cross-sectional descriptive survey design was used to collect data from 522 consenting adult Covid-19 survivors in Abuja (274) and Lagos (248), Nigeria, using a self-developed, close-ended and validated questionnaire called the Lifestyle-related Factors in Covid-19 Questionnaire (LFC-19 Questionnaire) through a multistage sampling technique. Descriptive and inferential statistical analysis was done using the Statistical Package for Social Science (SPSS) with P value set at ≤ 0.05. Ethical approval was obtained for the study.

**Results:**

A significant number of Covid-19 Survivors were overweight/obese (67.8%) and had a history of physical inactivity (73.8%). A small proportion had premorbid chronic diseases (23.8%) as well as pre-existing lifestyle-related risk factors such as inadequate consumption of fruits (67.2%) and vegetables (60.0%) and physical inactivity (73.8%).

**Conclusion:**

This study revealed that most Covid-19 survivors residing in Lagos State and in Abuja capital city of Nigeria were either overweight or obese. This was due to physical inactivity, an unhealthy diet consisting of low fruit and vegetable consumption and poor sleep. Additionally, the study showed that patients’ perceptions of their risk factors were often inaccurate as it differed from what was measured. The findings from this study will assist public health professionals and clinicians in designing and implementing more effective Covid-19 management strategies that incorporate healthy lifestyle practices and lifestyle modifications and assist public health promotion and communication specialists in designing appropriate and evidence-based preventive messages.

**Supplementary Information:**

The online version contains supplementary material available at 10.1186/s12889-024-19502-w.

## What do we already know about this topic?

Studies have shown that Covid-19 is related with morbidity and mortality and its association with clinical and non-clinical features including pre-morbid chronic medical conditions such as hypertension and diabetes e.t.c, having an aggravated single or multiple risk factors [7, 10].

## How does your research contribute to the field?

This study provides insight into the prevalence of premorbid lifestyle-related chronic disease risk factors among Covid-19 survivors in Lagos State and Abuja capital city of Nigeria and contributes to the field of social health and perception studies, carried out to understand the opinions of our target population (Covid-19 Survivors) in line with lifestyle related risk factors (such as unhealthy diet, physical inactivity, and poor sleep) associated with Covid-19. It should deepen the ongoing conversation around the relationship between unhealthy lifestyle and the contraction COVID-19.

## What are your research implications for theory, practice, or policy?

Optimizing environmental hygiene, vaccination, and treatment of symptomatology were central to the prevention and treatment of Covid-19. This research suggests that some pre-morbid unhealthy lifestyle choices (such as inadequate consumption of fruits and vegetables, and physical inactivity) were significantly high among the study participants that contracted Covid-19. Thus, adding healthy living and lifestyle changes into the Covid-19 prevention and treatment strategies by policymakers, public health professionals, and clinicians might result in better outcomes. The findings that most Covid-19 survivors in Lagos state and Abuja capital city Nigeria had premorbid unhealthy lifestyles and the fact that they were not aware will assist public health professionals and clinicians in designing and implementing more effective Covid-19 management strategies that incorporate healthy lifestyle practices and lifestyle modifications and assist public health promotion and communication specialists in designing appropriate and evidence-based preventive messages. This could be achieved by incorporating lifestyle modification counselling and prescription into routine care for adults particularly obese adults.

## Introduction

Coronavirus disease 2019 (Covid-19) was first identified in medical taxonomy in late 2019. The disease was later confirmed to be caused by a novel coronavirus, severe acute respiratory syndrome coronavirus 2 (SARS-CoV-2), which was first described among the Chinese individuals in December 2019. It was suspected that the virus originated from contact with fresh animals from a Wuhan animal market in China. It has been consistently proven that bats are the natural hosts of this virus. It may be mutated in intermediate hosts, such as the Malayan pangolin (which shares 91% nucleotide identity), from which it infects humans [1–4]. The virus has since spread globally, with devastating consequences for public health and the global economy. Globally, as of 19th April 2023, there were 763,740,140 confirmed cases of Covid-19, including 6,908,554 deaths, reported to the World Health Organisation (WHO) [5].

Although the index case of Covid-19 in Nigeria was not identified until the 28th of February 2020, the case of an Italian citizen who works in Nigeria and flew into Lagos, Southwest Nigeria, from Milan, three days prior, has since spread to all the states of the country [6, 7]. As of [19] April 2023, the country had more than 266,675 confirmed cases of Covid-19, with 3,155 deaths reported to the WHO [8]. The disease has taken its toll on the lives of citizens in various areas, including economic hardship. The nation was forced to undergo a complete shutdown at one point, leaving numerous businesses reeling from the devastating blow of massive income losses, ultimately resulting in the downfall of some unfortunate enterprises. Even three years after the first index case was reported in Nigeria, the economic impact of the disease still existed.

To break the Covid-19 triangle of agent, environment, and host, global public health professionals responded by proposing different preventive measures and guidelines, such as physical distancing, increased personal and environmental hygiene, and face-mask-wearing. These measures alone have not been successful in curbing the spread of the virus, especially in situations where they are not consistently or correctly implemented. Clinicians also institute different treatment protocols, with varying success rates. The principal medicines used to treat Covid-19 include remdesivir, hydroxychloroquine, lopinavir, interferon, and ivermectin. Unfortunately, an interim report by the World Health Organisation (WHO) suggested that the first four drugs are ineffective at preventing the clinical progression of the disease [9]. Great strides have been made in the attempt to vaccinate global populations against Covid-19, but as of 19 March 2023, only a total of 116,606,863 vaccine doses were administered in Nigeria [8]. Until more definitive treatment protocols are freely available and more people are vaccinated for Covid-19 worldwide, scientists must continue to research and develop more efficient evidence-based preventive and treatment measures against the disease [5].

Studies over the last three years of this pandemic have shown some of the protective and risk factors for contracting the disease as well as prognosticating clinical progression and severity of illness. Age and preexisting comorbidities such as hypertension, cardiovascular disease, chronic obstructive pulmonary disease, and obesity have been shown to play a significant role, with the disease affecting adults more than children and all ages with comorbidities having a higher risk than those in the same age bracket without those conditions [10].

Several studies have suggested that lifestyle-related risk factors for chronic diseases may also be associated with increased susceptibility to contracting SARS-CoV-2 and may further play a significant role in the clinical progression of Covid-19. Some of the suspected associated lifestyle choices are unhealthy diets (particularly diets that are low in vitamin C, D, and zinc), physical inactivity with poor exposure to sunlight, and tobacco use [11].

Studies have also shown that by weakening the immune system and causing systemic chronic inflammation, some lifestyle-related intermediate risk factors for cardiometabolic diseases, can increase the risk of developing or having a severe form of Covid-19. These factors include obesity, high blood pressure, high blood glucose, and high blood cholesterol [11–13]. It stands to reason that if having these comorbidities can predispose individuals to contracting COVID-19 or being very ill from it, then preventing the development of or adequately managing or even reversing these comorbidities could also prevent the contraction of the virus.

Many of these comorbidities, on their own, have been extensively studied with preventive and therapeutic regimens outlined and firmly rooted in lifestyle modification measures [14]. Thus, some studies have further suggested that optimizing lifestyles through the deliberate control of these related risk factors through healthy lifestyle practices and the right perception will likely reduce susceptibility to Covid-19 and ultimately reduce disease incidence and fatalities [15].

There are no known local or regional studies that have extensively investigated the prevalence of these suspected associated premorbid lifestyle choices and factors among confirmed Covid-19 patients. Additionally, no known well-designed international studies have extensively investigated the prevalence of several other emerging chronic disease risk factors, such as inadequate sleep duration, in confirmed Covid-19 patients. The perceptions that confirmed Covid-19 patients have of the role that the identified lifestyle-related risk factors might play in their contraction of the SARS-CoV-2 virus and the severity of Covid-19 have also not been substantially investigated. One notable principle in lifestyle medicine is patient autonomy. The patient will be in charge of their health outcomes with the support of the health care provider. This approach is so empowering and can be effective only when the patient realizes in the first place what the problem is and when they can make the difference, i.e., perception [16]. Consequently, this study investigated the prevalence and prevalence of premorbid lifestyle-related risk factors among Covid-19 survivors in Lagos state and Abuja capital city of Nigeria, to hopefully deepen the body of knowledge on Covid-19.

This study aimed to investigate the prevalence of premorbid lifestyle-related risk factors, such as unhealthy diets, physical inactivity, tobacco use, alcohol abuse, inadequate sleep duration, and medical history of high blood pressure, high blood glucose, high blood cholesterol, hypothyroidism or hyperthyroidism, asthma, and chronic obstructive pulmonary disease (COPD), among confirmed Covid-19 survivors in Lagos state and Abuja capital city of Nigeria. We also sought to examine the possible role that the identified lifestyle-related risk factors might have played in the contraction and severity of SARS-CoV-2 among confirmed Covid-19 survivors in Lagos state and Abuja capital city of Nigeria .

## Methodology

### Study setting

The study was conducted in Lagos State and Abuja capital city of Nigeria, the administrative and commercial capitals of Nigeria, respectively. Both cities have had a fair share of the Covid-19 pandemic with Lagos state and FCT ranked as the states with the highest and second highest confirmed cases respectively.

There were a total of 29,187 confirmed cases with 28,814 survivors in Abuja as at 28th August 2022, ranking second among the number of confirmed Covid-19 cases in the country [17]. Abuja, the capital of Nigeria, is located in northcentral Nigeria and covers an area of 1,476 square km a population of 1,236,000 in 2022 [18]. It is home to all the major ethnic groups in Nigeria with majority of its workforce being government workers, and its biggest healthcare facility is the National Hospital, Abuja.

Lagos state had the highest number of confirmed Covid-19 cases in the country with 103,253 confirmed cases and 101,977 survivors as at 28th August 2022 [17]. Lagos is located in southwest Nigeria and had a population of 15,388,000 in 2022 [18]. It is the commercial capital of Nigeria and was the administrative capital until 1991 when it was moved to Abuja [19].

Their geographical location and cosmopolitan nature mean that most ethnic groups and other sociodemographic variables in Nigeria are largely represented in both cities. They also house the headquarters complexes of many public and private organizations in Nigeria, including the two largest and busiest international airports in the country, thereby attracting hundreds of travellers, both nationally and internationally, on a day-to-day basis. Frequent travel and interaction have been well-documented as risk factors for the spread of the coronavirus. All these observations confirmed that Abuja and Lagos were ideal locations for the study.

### Study design

A cross-sectional descriptive survey was used for the study.

### Study population

This study was conducted among adults 18 years and above who resided in Lagos state and Abuja capital city of Nigeria and were presently or previously confirmed to be Covid-19 positive.

**Inclusion criteria** for identifying eligible participants included the following:

1. People who tested positive for Covid-19 at a government-approved facility at any time during the pandemic.
2. People living in Abuja and Lagos, Nigeria.
3. People aged 18 years and older were included.
4. People who signed the consent form were included in the study.

### Exclusion criteria

The following participants were excluded from the study.

1. Those who did not provide complete information.
2. Those who could not fill out the form due to cognitive impairment or any other form of disability.

### Sample size determination

The sample size for this study was 251 participants who tested positive for COVID-19 in Nigeria. This was calculated using the Research Sample Size Formula by Cochran in 1977: n = z^2^ pq/d^2^. where n = the desired sample size when the study population is greater than 10,000 and z = the standard normal deviation, usually set at 1.96, which corresponds to a 95% confidence interval (*p*) = prevalence from previous studies. As no previous studies could be found, 50% was used (*p* = 0.5) q = 1 - *p* 1–0.5 = 0.5 d = degree of accuracy desired, usually set at 0.05. Therefore, n = (1.96^2^ × 0.5 × 0.5)/0.05^2^*n* = 0.656/0.0025 *n* = 262. Using the formula, the actual sample size was 262. With the addition of a 10% attrition rate, the sample size was determined to be 262 + 26 = 288 to account for factors such as missing questionnaires and incorrectly/incompletely filled questionnaires.

### Sampling technique

A multistage sampling technique was used for the study. In the first stage, an advertisement was made through social media, and a purposive sampling technique was used to select participants who were interested in the study. In the second stage, a simple random sampling technique was used to select the study population from the people who consented to participate in the study and met the inclusion and exclusion criteria. Here, the email addresses of all the people who showed interest in the study were placed in black (non-transparent) bags, and email addresses were randomly selected manually from the bags without replacement until the desired sample size was achieved. The people who owned the selected email addresses were selected as the study participants.

Patients who presented to the Family Medicine Clinics of the National Hospital, Abuja, Wuse District Hospital, Abuja, Lagos University Teaching Hospital, (LUTH), Idi-Araba, Lagos and Lagos State University Teaching Hospital (LASUTH) Ikeja Lagos were also consecutively recruited until the desired sample size was achieved.

#### Study instrument

A self-developed, close-ended and validated questionnaire called the Lifestyle-related Factors in Covid-19 Questionnaire (LFC-19 Questionnaire), developed for this study, was used for data collection (Attached as Supplementary file). The instrument has 4 sections: A (demography), B (medical history), C (premorbid lifestyle history), and D (perception of lifestyle and Covid-19).

#### Validity of the research instrument

To validate the self-developed research instrument, a draft of the instrument was made available for evaluation to experts in the fields of lifestyle medicine and public health as well as other experts in related fields of internal medicine, biostatistics, epidemiology, nutrition, and sleep medicine. The instruments also assessed content and construct validity to ensure coverage of all the areas considered in the study. All necessary corrections, modifications, and suggestions were incorporated into the instrument before it was administered.

#### Reliability of the research instrument

To determine the reliability of the self-developed instrument, it was administered to 20 known confirmed Covid-19 Nigerians. The data collected thereafter were coded, entered into SPSS statistical software and subjected to Cronbach’s alpha coefficient analysis to determine the internal consistency and reliability of the questionnaire. A Cronbach’s alpha score of 0.86 was obtained. All the appropriate observations and changes were incorporated into the main study before it commenced.

#### Procedure for data collection

The instrument was administered, and the data were collected online and offline. An advertisement was made for the study through different social media (LinkedIn, Facebook, WhatsApp, Instagram, and Twitter) platforms. Interested participants were required to follow a Google link through which the consent form and questionnaire were made available. The first page of the online questionnaire was the consent page. Participants were only able to proceed after consenting to participate. The BMI was determined by asking the patients to present to any of the above study centres closest to them and their weights and heights were measured. This was then used to determine BMI.

Adult patients who presented to the Family Medicine Clinic of the selected hospitals, consented to the study and met the selection criteria were also administered the questionnaire. Their weights and heights were measured and BMI determined.

### Study variables

The independent variable was the study location while the outcome (dependent) variable was the pre morbid lifestyle choices among the study participants.

### Operational definition of terms

1. Alcohol abuse was defined as the consumption of more than 2 standard bottles of alcoholic beverages daily by the participants.
2. Inadequate sleep duration: This means the inability of the participants to engage in a minimum of 7 h of uninterrupted sleep every night.
3. Lifestyle-Related Risk Factors: These were factors directly or indirectly resulting from lifestyle choices and increase the likelihood of participants developing chronic noncommunicable diseases compared to others.
4. Perception: This means the way the participants regarded, understood, or interpreted the association between Covid-19 and the dependent variables.
5. Premorbid lifestyle: This variable refers to the behavioural and lifestyle choices that the participants usually make before they were diagnosed with a positive test for Covid-19.
6. Prevalence: This variable is the number of dependent variables that are present in the participants.
7. Physical Inactivity: This refers to the inability of the participants to engage in moderate to intense physical activities (such as brisk walking, gardening, etc.) for at least 30 min a day, 5 days a week or 150 min a week; OR, the inability of the participants to engage in vigorous physical activities (such as running, playing football, etc.) for at least 20 min a day or 120 min a week.
8. Unhealthy diet: This refers to the inability of the participants to consume fruits and vegetables for a minimum of 5 days a week and the inability to use olive oil/canola oil to prepare their household food on most days.

#### Data analysis

The data collected from participants were subjected to both descriptive and inferential statistics. The Statistical Package for Social Science (SPSS) for Windows, version 15 software (SPSS, Inc., Chicago, IL), was used to analyse the data. Sociodemographic characteristics, premorbid lifestyle choices and Respondents’ Perception of Association between Unhealthy Lifestyle Choices and Covid-19 Disease were analysed descriptively and reported using frequency count, percentage, and bar charts. The data were expressed as the mean ± SD. The chi-square test was used for inferential statistics to test for association between location of respondents and unhealthy lifestyle choices as well as association between unhealthy lifestyle choices and BMI. Multinomial logistic regression was used to further test for the association between unhealthy lifestyle choices and BMI. P value ≤ 0.05 was considered statistically significant at 95% confidence interval.

## Results

Of the 527 respondents who participated in the study, 5 were suspected of having not yet been confirmed (and so were dropped from all analyses).

**Fig. 1:**
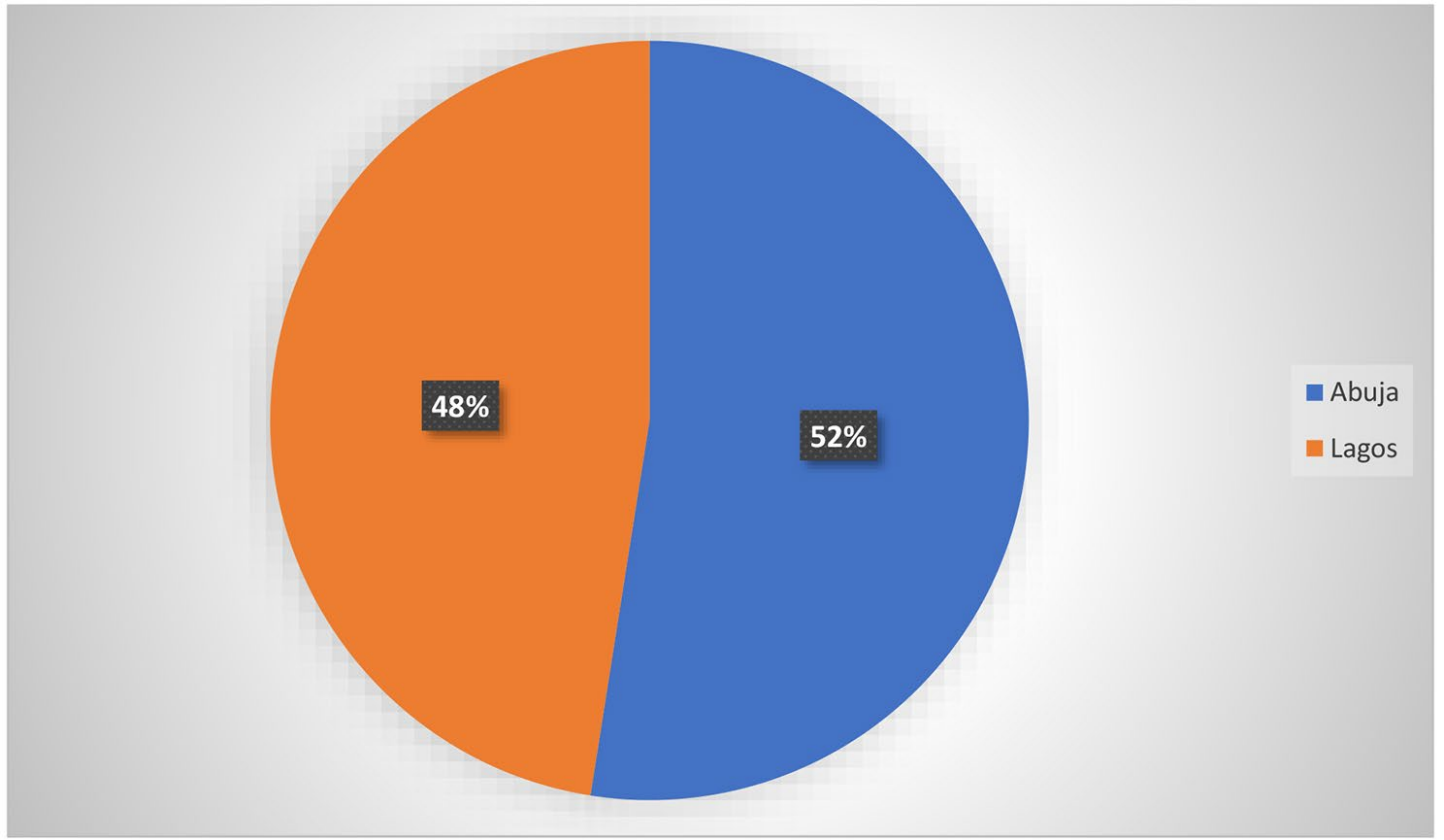
Location of Respondents (*n* = 522)

A total of 274 (52.5%) of the respondents resided in Abuja, while the remaining 248 (47.5%) resided in Lagos (Fig. 1).

The sociodemographic characteristics of the 522 respondents who met the selection criteria and participated in the study are outlined in Table 1. The respondents were mostly males (289, 55.4%), with ages ranging from 16 to 75 years and the majority of them were in their sixth decade of life (180, 34.5%), were married (305, 58.4%) and had a tertiary level of education (300, 57.5%). There was no statistically significant difference between the sociodemographic characteristics of the respondents in both Lagos state and Abuja capital city of Nigeria.

**Table 1:** Sociodemographic characteristics of the respondents (*n* = 522)

Variable	Frequency (Per cent) (*n* = 522)			χ [2]	*P* Value
	**Abuja** **274 (52.5)**	**Lagos** **248 (47.5)**	**Total** **522 (100)**		
Gender				0.090	0.765
Male	150 (54.7)	139 (56.0)	289 (55.4)		
Female	124 (45.3)	109 (44.0)	233 (44.6)		
Age in years				5.545	0.236
Below 30	67 (24.5)	56 (22.6)	123 (23.5)		
31–40	30 (10.9)	28 (11.3)	58 (11.1)		
41–50	70 (25.5)	61 (24.6)	131 (25.1)		
51–60	86 (31.4)	94 (37.9)	180 (34.5)		
Above 60	21 (7.7)	9 (3.6)	30 (5.8)		
Marital status				4.603	0.330
Single	69 (25.2)	61 (24.6)	130 (24.9)		
Married	153 (55.8)	152 (61.3)	305 (58.4)		
Divorced	15 (5.5)	7 (2.8)	22 (4.2)		
Separated	20 (7.3)	19 (7.7)	39 (7.5)		
Widowed	17 (6.2)	9 (3.6)	26 (5.0)		
Highest level of education completed				7.019	0.135
No formal education	7 (2.6)	4 (1.6)	11 (2.1)		
Primary school	16 (5.8)	7 (2.8)	23 (4.4)		
Secondary school	55 (20.1)	38 (15.3)	93 (17.8)		
OND/HND/First degree	145 (52.9)	155 (62.5)	300 (57.5)		
Postgraduate degree	51 (18.6)	44 (17.8)	95 (18.2)		

**Fig. 2:**
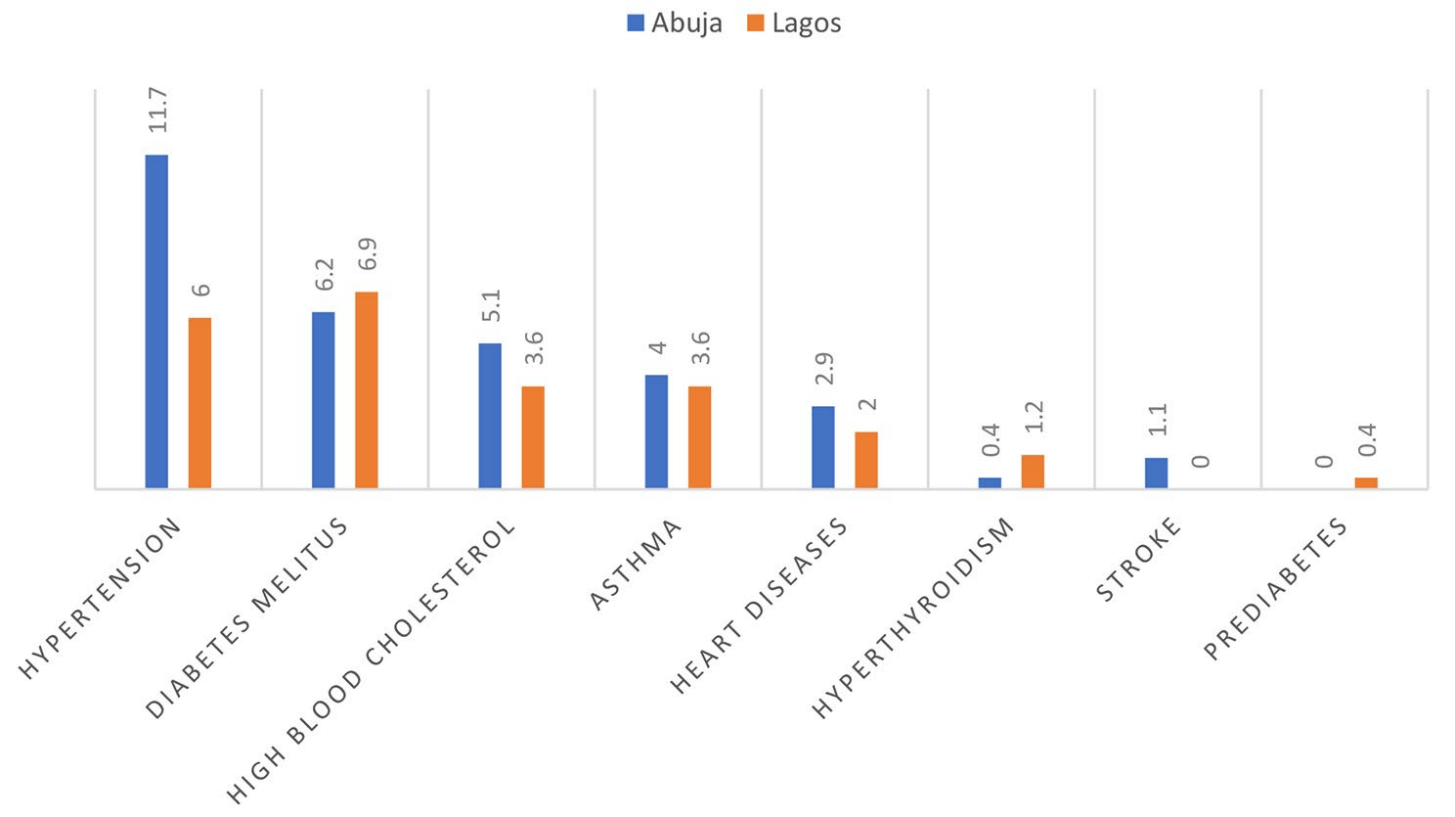
Premorbid medical history of chronic diseases (*n* = 522). * Multiple response

Premorbid conditions were not common, as less than a quarter 124 (23.8%) of the respondents reported a history of a chronic medical condition. The most prevalent premorbid condition was hypertension, with majority of the patients with hypertension residing in Abuja 32 (11.6%) compared to Lagos state 15 (6.0%). This was followed by diabetes with residents in Abuja 17 (6.2%) and Lagos State 17 (6.9%). Hyperthyroidism, stroke and prediabetes were rare, with less than 1% of respondents reporting a history of each of these conditions (Fig. 2).

Over two-thirds (354; 67.8%) of the respondents were overweight/obese, while approximately 30% had a normal BMI. However, a large majority (437; 83.7%) of the respondents described their weight as normal. Only 77 (14.8%) of the participants described their weight as overweight/obese. There was poor agreement between the actual BMI of the respondents and their perception of weight (kappa = 0.099). This was more the case for respondents who said they were underweight or of normal weight, as only 2 (25.0%) respondents claimed to be underweight and 143 (32.7%) of those who said they were of normal weight were underweight. However, 70 (90.9%) of those who said they were overweight or obese did so (Table 2).

**Table 2:** Comparison of respondents’ actual BMI and their perceived weight (*n* = 522)

	Actual BMI category			
Perceived Weight	Underweight *n* (%)	Normal *n* (%)	Overweight/obese *n* (%)	Total *N* (%)
Underweight	2 (25.0)	4 (50.0)	2 (25.0)	8 (100.0)
Normal	12 (2.8)	143 (32.7)	282 (64.5)	437 (100.0)
Overweight/obese	0 (0.0)	7 (9.1)	70 (90.9)	77 (100.0)
Total	14	154	354	522

Kappa = 0.099

Table 3 shows the premorbid lifestyle patterns of the respondents. Most of the respondents (351 [67.2%] ate fresh fruits for less than 5 days a week], 313 (60.0%) ate vegetables for less than 5 days a week, 486 (93.1%) engaged in moderate exercise for less than 5 days a week, 409 (78.4%) engaged in vigorous exercise for less than 3 days a week, and 359 (68.8%) slept for less than 7 h a day. However, only 36 (6.9%) took tobacco, and 35 (6.7%) drank more than 2 standard alcoholic drinks in the preceding month.

**Table 3:** Premorbid lifestyle choices (*n* = 522)

Variable	Frequency (*n* = 522)	Per cent (%)
Frequency of eating fresh fruits (days per week)		
< 5	351	67.2
≥ 5	171	32.8
Frequency of eating of vegetables (days per week)		
< 5	313	60.0
≥ 5	209	40.0
Oil or fat most used for food preparation in households*		
Palm oil	435	83.3
Vegetable oil	161	30.8
Groundnut oil	44	8.4
Olive/Canola oil	30	5.8
Lard	29	5.6
Butter/Margarine	27	5.2
Rapseed and Canola	1	0.2
Soya	1	0.2
None in particular	3	0.6
Intake of tobacco products		
Yes	36	6.9
No	486	93.1
Drinking more than 2 standard alcoholic drinks in the past 30 days		
Yes	35	6.7
No	206	39.5
Never drink alcohol	281	53.8
Frequency of moderate-intensity physical activities (days per week)		
< 5	385	73.8
≥ 5	137	26.2
Frequency of vigorous-intensity physical activities (days per week)		
< 3	409	78.4
≥ 3	113	21.6
Duration of night sleep on most days (hours)		
< 7	359	68.8
≥ 7	146	28.0
Don’t know	17	3.3

*Multiple response

Participants residing in Lagos were more likely to have unhealthy eating habits related to fruits and vegetables than were those residing in Abuja. While more than 70% of the Lagos residents ate vegetables less than 5 days a week, only 48.9% of the Abuja residents had such a history (*p* < 0.001). Among the oils most commonly used for meal preparation, Abuja residents had significantly greater proportions than Lagos residents for all the different types of oils, except palm oil, which was not significantly different (80.3% vs. 86.7%, *p* = 0.050). Abuja residents were also found to have a significantly greater proportion of tobacco users and heavy drinkers of alcohol than Lagos residents. Unhealthy exercise and sleep habits were more prevalent in Lagos residents than in Abuja residents (Table 4).

**Table 4:** Location and unhealthy lifestyle choices (*n* = 522)

Variable	Location		Z	*P*
	Abuja *N* = 274 *n* (%)	Lagos *N* = 248 *n* (%)		
Eats fresh fruits < 5 days a week	165 (60.2)	186 (75.0)	-3.59	< 0.001
Eats vegetables < 5 days a week	134 (48.9)	179 (72.2)	-5.42	< 0.001
Palm oil is most often used	220 (80.3)	215 (86.7)	-1.96	0.050
Vegetable oil is most often used	109 (39.8)	52 (21.0)	4.65	< 0.001
Groundnut oil is most often used	36 (13.1)	8 (3.2)	4.07	< 0.001
Olive/Canola oil is most often used	23 (8.4)	7 (2.8)	2.73	0.006
Lard most often used	24 (8.8)	5 (2.0)	3.36	0.001
Butter/margarine is most often used	23 (8.4)	4 (1.6)	3.49	< 0.001
Intake of tobacco products	25 (9.1)	11 (4.4)	2.11	0.035
Drinking more than 2 standard alcoholic drinks in the past 30 days	22 (8.0)	13 (5.2)	1.27	0.204
Do moderate-intensity physical activities < 5 days a week	191 (69.7)	194 (78.2)	-2.21	0.027
Does vigorous-intensity physical activities < 3 days a week	205 (74.8)	204 (82.3)	-2.06	0.039
Sleep < 7 h at night on most days	162 (59.1)	197 (79.4)	-5.00	< 0.001

The respondents’ perceptions of the association between unhealthy lifestyle choices and COVID-19 disease are shown in Table 5. Over half of the respondents believed that persons with underlying chronic diseases (such as hypertension and diabetes mellitus) were at a greater risk of developing a severe form of COVID-19 (278 [53.3%) and dying from the disease (313 [60.0%) than people who did not have any of these diseases were.

**Table 5:** Respondents’ Perception of Association between Unhealthy Lifestyle choices and Covid-19 Disease (*n* = 522)

Statement	Strongly disagree *n* (%)	Disagree *n* (%)	Neutral *n* (%)	Agree *n* (%)	Strongly agree *n* (%)
Regular consumption of fruits and vegetables can protect a person from contracting the Covid-19 virus.	6 (1.2)	23 (4.4)	96 (18.4)	189 (36.2)	208 (39.8)
Regular consumption of fried and processed foods/drinks like meat pies, donuts, cakes, and fizzy drinks can increase the chances of a person contracting the Covid-19 virus.	32 (6.1)	226 (43.3)	69 (13.2)	75 (14.4)	120 (23.0)
Some foods (like soy milk and mushrooms) can prevent a person from contracting the Covid-19 virus.	71 (13.6)	184 (35.3)	74 (14.2)	79 (15.1)	114 (21.8)
Some fish (like tuna, mackerel, herring, and salmon) can prevent a person from contracting the Covid-19 virus.	84 (16.1)	178 (34.1)	68 (13.0)	82 (15.7)	110 (21.1)
Regular exposure to sunlight can reduce a person’s chances of contracting the Covid-19 virus.	24 (4.6)	55 (10.5)	86 (16.5)	201 (38.5)	156 (29.9)
Persons who are overweight or obese are at higher risk of contracting the Covid-19 virus compared to persons with normal weight.	4 (0.8)	18 (3.5)	44 (8.4)	211 (40.4)	245 (46.9)
Persons with underlying chronic diseases (like hypertension and diabetes mellitus) are at a higher risk of developing a severe form of Covid-19 compared to persons who do not have any of these diseases.	2 (0.4)	10 (1.9)	29 (5.6)	203 (38.9)	278 (53.3)
Persons with underlying chronic diseases (like hypertension and diabetes mellitus) are at a higher risk of dying from Covid-19 compared to persons who do not have any of these diseases.	1 (0.2)	9 (1.7)	21 (4.0)	178 (34.1)	313 (60.0)

The association between lifestyle choices and BMI was determined using chi square test. More than three-quarters of those who engaged in moderate-intensity physical activity less than 3 days per week were overweight/obese, whereas 43% of those engaged in moderate-intensity physical activity 3 days or more per week were overweight/obese (*p* < 0.001). Similar findings were observed for vigorous-intensity physical activity (Table 6).

**Table 6:** Unhealthy lifestyle choices and BMI (*n* = 522)

Variable	Underweight *n* (%)	Normal *n* (%)	Overweight/obese *n* (%)	χ [2]	*P*
**Frequency of eating fresh fruits (days per week)**					
< 5	8 (2.3)	91 (25.9)	252 (71.8)	7.794	0.020
≥ 5	6 (3.5)	63 (36.8)	102 (59.7)		
**Frequency of eating of vegetables (days per week)**					
< 5	3 (1.0)	76 (24.3)	234 (74.8)	21.440	< 0.001
≥ 5	11 (5.3)	78 (37.3)	120 (57.4)		
**Intake of tobacco products**					
Yes	0 (0.0)	13 (36.1)	23 (63.9)	1.698	0.428
No	14 (2.9)	141 (29.0)	331 (68.1)		
**Drinking more than 2 standard alcoholic drinks in the past 30 days**					
Yes	1 (2.9)	8 (22.9)	26 (74.3)	22.480	< 0.001
No	2 (1.0)	42 (20.4)	162 (78.6)		
Never drink alcohol	11 (3.9)	104 (37.0)	166 (59.1)		
**Frequency of moderate-intensity physical activities (days per week)**					
< 5	4 (1.0)	86 (22.3)	295 (76.6)	57.066	< 0.001
≥ 5	10 (7.3)	68 (49.6)	59 (43.1)		
**Frequency of vigorous-intensity physical activities (days per week)**					
< 3	5 (1.2)	94 (23.0)	310 (75.8)	59.957	< 0.001
≥ 3	9 (8.0)	60 (53.1)	44 (38.9)		
**Duration of night sleep on most days (hours)**					
< 7	7 (1.9)	80 (22.3)	272 (75.8)	35.717	< 0.001
≥ 7	6 (4.1)	69 (47.3)	71 (48.6)		
Don’t know	1 (5.9)	5 (29.4)	11 (64.7)		

Table 7 shows the results of a multinomial logistic regression model indicating the relationships between unhealthy lifestyle choices and BMI. Those who engaged in moderate-intensity physical activity less than 5 days a week were 3.58 times more likely to be overweight/obese than were those who had 5 or more days of moderate-intensity physical activity per week (aOR = 3.58; 95% CI = 2.25 to 5.69; *p* < 0.001). Similarly, the odds of being overweight or obese were also greater for individuals who were less frequently engaged in vigorous physical activity (aOR = 3.83; 95% CI = 2.34 to 6.26; *p* < 0.001).

**Table 7:** Multinomial logistic regression model showing relationships between unhealthy lifestyle choices and BMI

Variable	Crude OR (95% CI)	*P*	Adjusted OR* (95% CI)	*P*
**Frequency of eating fresh fruits (days per week)**				
< 5	0.92 (0.31 to 2.79)	0.008	1.53 (0.98 to 2.40)	0.062
≥ 5	1 (reference)		1 (reference)	
**Frequency of eating of vegetables (days per week)**				
< 5	2.00 (1.36 to 2.94)	< 0.001	1.65 (1.05 to 2.59)	0.029
≥ 5	1 (reference)		1 (reference)	
**Intake of tobacco products**				
Yes	0.75 (0.37 to 1.53)	0.434	0.86 (0.39 to 1.91)	0.706
No	1 (reference)		1 (reference)	
**Drinking more than 2 standard alcoholic drinks in the past 30 days**				
Yes	2.04 (0.89 to 4.67)	0.093	2.20 (0.90 to 5.39)	0.086
No	2.42 (1.59 to 3.67)	< 0.001	2.04 (1.28 to 3.25)	0.003
Never drink alcohol	1 (reference)		1 (reference)	
**Frequency of moderate-intensity physical activities (days per week)**				
< 5	3.95 (2.59 to 6.04)	< 0.001	3.58 (2.25 to 5.69)	< 0.001
≥ 5	1 (reference)		1 (reference)	
**Frequency of vigorous-intensity physical activities (days per week)**				
< 3	4.50 (2.86 to 7.07)	< 0.001	3.83 (2.34 to 6.26)	< 0.001
≥ 3	1 (reference)		1 (reference)	
**Duration of night sleep on most days (hours)**				
≥ 7	1 (reference)		1 (reference)	
< 7	3.30 (2.18 to 5.00)	< 0.001	2.78 (1.77 to 4.39)	< 0.001
Don’t know	2.14 (0.71 to 6.47)	0.179	2.58 (0.77 to 8.61)	0.124

OR = odds ratio (odds of having overweight or obese BMI/odds of having normal BMI)

*Adjusted for age, sex, marital status, education, and residence

## Discussion

This study was conducted to investigate the prevalence and perception of premorbid lifestyle-related risk factors among Covid-19 patients in Abuja and Lagos, Nigeria. The study showed that most respondents were males and patients in their sixth decade of life. These findings are similar to those of studies conducted in China, Italy and the USA, which revealed that Covid-19 infection affects more males and elderly people [12, 20]. Whereas the above studies revealed a greater prevalence of Covid-19 among patients in their 7th or older decade of life, this study revealed a greater prevalence of Covid-19 among patients in their 6th decade. This may be due to the greater life expectancy in these countries compared to Nigeria, where the life expectancy is currently 55 years [21].

The majority of the study participants did not have any premorbid medical conditions before developing Covid-19. However, of those who reported a premorbid condition, hypertension was the most commonly reported. This finding is contrary to findings from most studies where Covid-19 incidence was found to be greater in patients with premorbid conditions. The disease was also more serious in those patients and was associated with greater hospital admission and fatality [22–26]. The difference in the findings of this study compared with those of other studies may be because this study was conducted among survivors of Covid-19, and studies have shown that patients without comorbidities have a greater survival rate and better treatment outcomes [12, 26].

A large number of the Covid-19 patients investigated were overweight/obese. This finding is similar to findings from other studies in which lifestyle-related factors such as overweight and obesity were strongly associated with increased susceptibility to Covid-19 [27, 28]. The high prevalence of obesity observed in the study was further reflected in the lifestyle patterns of the respondents. The participants mostly lived a sedentary lifestyle (*p* < 0.001), ate unhealthy diets low in vegetables (*p* < 0.001) and did not have restorative sleep (*p* < 0.001). There is a need to increase the amount of physical activity and healthy eating among Nigerians, as unhealthy lifestyles also predispose patients to noncommunicable diseases (NCDs), such as hypertension, cardiovascular diseases, diabetes mellitus, kidney diseases and some cancers [29–31].

However, despite the high prevalence of overweight and obesity found in this study, most respondents perceived their weight as normal. This has serious implications for action. This explains why obesity is a major problem in society, for which minimal progress has been made in addressing it. Most of the people who were obese thought their weight was normal and therefore did not see any reason to reduce it. Efforts should therefore be made to create awareness as to what constitutes a normal weight and obesity. Most Nigerians perceive being overweight as normal and evidence of a good life. This should change to prevent the associated sequelae with obesity highlighted above.

Although smoking is another lifestyle-related factor that has been linked to increased susceptibility to and worsening outcomes from Covid-19 infection [12], this study revealed that most of the patients studied smoked or drank alcohol. This is quite impressive. This may have resulted from the massive campaigns against alcohol and tobacco and the deliberate policies by the Nigerian government to check excessive alcohol consumption and smoking, particularly in major cities such as Abuja and Lagos, where the study was conducted. Such policies include warning of the dangers of tobacco smoking on packs, high taxes on drinks and cigarettes and enlightenment activities on the dangers of alcohol and cigarettes.

Most respondents believed that regular consumption of fruits and vegetables, regular exposure to sunlight, not being obese, and not having an underlying chronic medical condition were protective against Covid-19 infection. However, they mostly did not believe that regular consumption of fried and processed food or nonconsumption of some foods, such as soymilk and fish, increased their likelihood of contracting the disease. Changing their negative perceptions and reaffirming their positive perceptions will help them prevent and manage not only Covid-19 but also other lifestyle-related conditions, such as NCDs. This can be achieved through creating public awareness of the health benefits of lifestyle modifications.

### Strengths and limitations of the study

The study evaluated the premorbid lifestyle pattern of Covid-19 survivors in Lagos State and Abuja capital city of Nigeria. This will provide an insight into the role of lifestyle choices in diseases.

The cross-sectional nature of the study enabled the authors determine the association between the locations and lifestyle choices highlighting the role of geographical locations in determining lifestyle choices in Nigeria.

This study was conducted among patients who claimed to be presently confirmed to be Covid-19 positive or who had developed the disease in the past. Since the researchers did not have access to the documented evidence of the participants’ claims, it was impossible to confirm their claims. To control this possible limitation, the researchers solicited the support of medical professionals (those based in Abuja and Lagos) to administer the study questionnaire to confirmed Covid-19 patients. Additionally, the researchers demanded in the questionnaire that the respondents state the medical facility that confirmed their Covid-19 status and, if possible, the date of confirmation. Any questionnaires with unsatisfactory responses were discarded.

Some people may not have been interested in completing a questionnaire for fear of stigmatization. To control this possible limitation, in addition to ensuring confidentiality, the names and addresses of the participants were needed. Additionally, as a form of incentive, the researchers gave health tips on how to strengthen personal immunity in the treatment and recovery from Covid-19 to all participants after they had completed the questionnaire in the form of an eBook authored by the researchers.

The sample may not be truly representative of all confirmed Covid-19 patients in Nigeria, as the study was conducted in Lagos and Abuja only. Although we cannot generalize the findings from this study to the whole country, we can still rely on the findings to conclude, as these two cities had the highest number of cases in the country and their residents were people from all over the country, with Abuja and Lagos being the administrative and economic capitals of Nigeria, respectively.

#### Implications of this study

The findings from this research are expected to contribute significantly to the related knowledge in the following areas.

1. This study will assist stakeholders in gaining insight into the prevalence of premorbid lifestyle-related chronic disease risk factors among Covid-19-positive patients in Nigeria.
2. These findings further strengthen the present scientific position that some premorbid lifestyle-related chronic disease risk factors have a negative role in the development of Covid-19.
3. The findings will assist public health professionals and clinicians in designing and implementing more effective Covid-19 management strategies that incorporate healthy lifestyle practices and lifestyle modifications.
4. An in-depth understanding of the possible role that the identified lifestyle-related risk factors play in the contraction of SARS-CoV-1 in Covid-2-positive patients could further assist public health promotion and communication specialists in designing appropriate and evidence-based preventive messages.

## Conclusions

The majority of Covid-19 survivors in Lagos and Abuja, Nigeria, were overweight or obese. This was due to unhealthy lifestyles, such as physical inactivity, unhealthy diets consisting of low fruit and vegetable consumption and poor sleep. Measures should be taken to improve lifestyle habits, such as increased physical activity, healthy diet and restorative sleep, as these measures have been shown to reduce the risk of Covid-19 and NCDs and improve outcomes.

### Electronic supplementary material

Below is the link to the electronic supplementary material.

Supplementary Material 1

## Data Availability

All data generated or analysed during this study are included in this published article.
