# Correction: Prevalence and perception of pre-morbid lifestyle-related risk factors among covid-19 survivors in Lagos state and Abuja capital city of Nigeria

**DOI:** 10.1186/s12889-024-19527-1

**Published:** 2024-08-06

**Authors:** Ifeoma N Monye, Tijani Idris Ahmad Oseni, Moyosore T. Makinde, Abiodun B. Adelowo, Safiya Yahaya-Kongoila, Marvellous C. Njoku-Adeleke, Aramide Oteju, Samba Nyirenda, Temitayo O. Elebiyo, Ijeoma Judith Dozie, Chinasa T. Ugwuegbulem-Amadi

**Affiliations:** 1 Society of Lifestyle Medicine of Nigeria (SOLONg), Lagos, Nigeria; 2 Brookfield Clinics Centre for Lifestyle Medicine, Department of Family Medicine, National Hospital, Abuja, Nigeria; 3 Department of Family Medicine, Edo State University, Edo State University Teaching Hospital, Uzairue, Auchi, Edo State Nigeria; 4 Department of Family Medicine, Lagos State University Teaching Hospital, Ikeja, Lagos Nigeria; 5 Niger Delta Power Holding Company, Abuja, Nigeria; 6 Department of Paediatrics, Wuse District Hospital, Abuja, Nigeria; 7 Department of Family Medicine, Lagos University Teaching Hospital, Lagos, Nigeria; 8 Sarai Holistic Care, Francistown, Botswana; 9 Department of Family Medicine, University of Benin Teaching Hospital, Benin City, Nigeria; 10 Department of Family Medicine, Federal University Teaching Hospital, Owerri, Imo State Nigeria; 11 Ariella Health and Fitness Limited/Queen of Peace Hospital, Port Harcourt, Rivers State Nigeria

**Correction to: BMC Public Health (2024) 24:1918**

10.1186/s12889-024-19502-w

The original publication of this article contained an incorrect author name. The incorrect and correct information is listed in this correction article. The original article has been updated.

Incorrect:

Ify Monye

Correct:

Ifeoma N Monye

