# Supplementary material for: Prevalence and perception of pre-morbid lifestyle-related risk factors among covid-19 survivors in Lagos state and Abuja capital city of Nigeria
Source: BMC Public Health. 2024 Jul 17;24:1918. doi: 10.1186/s12889-024-19502-w (PMC11256610; doi:10.1186/s12889-024-19502-w)
Supplement: Supplementary file 1 — Supplementary Material 1 [file 12889_2024_19502_MOESM1_ESM.docx]

APPENDIX I

LIFESTYLE-RELATED FACTORS IN COVID-19 QUESTIONNAIRE

(LFC-19 Questionnaire)

SECTION A: DEMOGRAPHY

1. Gender: (a) Male [ ] (b) Female [ ]

2. Age Range (Years): (a) < 18 [ ] (b) 18 - 30[ ] (c) 31 - 40 [ ] (d) 41- 50[ ] (e) 51- 60[ ] (f) > 60[ ]

3. Marital Status: (a) Single [ ] (b) Married [ ] (c) Separated [ ] (d) Divorced [ ] (e) Widowed [ ]

4. What is the highest level of education you have completed? (a) No formal education [ ] (b) Primary school completed [ ] (c) Secondary school completed [ ] (d) OND/HND/First Degree completed [ ] (e) Post graduate degree completed [ ]

5. Do you reside in Abuja, Nigeria? (a) Yes [ ] (b) No [ ]

SECTION B: MEDICAL HISTORY

6. Have you ever been told by health professionals that you have COVID-19? (a) Yes, there is a suspicion, but it has not been confirmed by test [ ] (b) Yes, I have had a positive laboratory test [ ] (c) No, I have never been diagnosed with COVID-19 [ ]

7. If ‘Yes’ to any of the above questions, kindly name the medical facility that made the diagnosis, ………………………………………………………………………………………

8. Have you ever been told by health professionals that you have any of the followings: (a) High Blood Pressure/Hypertension [ ] (b) Stroke [ ] (c) High Blood Sugar/Diabetes mellitus [ ] (d) Heart Diseases/Heart Attack [ ] (e) High Blood Cholesterol [ ] (f) Asthma [ ] (g) Hyperthyroidism [ ] (h) Hypothyroidism [ ] (i) No to any of the stated diseases [ ]

SECTION C: PRE-MORBID LIFESTYLE HISTORY

For the people that are presently or were formally diagnosed positive for COVID-19, kindly recall back to the period before the diagnosis was made, which of the following do you think were true:

9. What is your present weight: …………..

10. What is your present height? ……………………

11. How would you describe your weight? (a) Underweight [ ] (b) Normal [ ] (c) Overweight/Obese [ ]

12. In a typical week, on how many days do you eat fruits (not processed)? (a) < 5days [ ] (b) ≥ 5 days [ ]

13. In a typical week, on how many days do you eat vegetables? (a) < 5days [ ] (b) ≥ 5 days [ ]

14. What type of oil or fat is most often used for meal preparation in your household? (Tick as many as applicable) (a) Olive/Canola oil [ ] (b) Ground oil [ ] (c) Palm oil [ ] (d) Fats/oil from meats [ ] (e) Butter/Margarine [ ] (f) None in particular [ ] (g) Others (Please specify) ………………………

15. Do you take any tobacco products, such as cigarettes, cigars, pipes or snuff? (a) Yes [ ] (b) No [ ]

16. During the past 30 days, when you drank alcohol, on average, do you drink more than 2 standard alcoholic drinks (Beer, Wine, Spirit, local Gin) on a day or during one drinking occasion? Kindly note: 1 standard alcoholic drink is equal to about half a pint (a pint can be equivalent to a sachet of pure water) of ordinary strength beer or lager e.g., Guinness, Gulder, Star etc. (3-4% alcohol by volume), or a small pub measure (25 ml) of spirits (40% alcohol by volume), or a standard pub measure (50 ml) of fortified wine such as sherry or port (20% alcohol by volume). (a) Yes [ ] (b) No [ ] (c) I don’t drink alcohol [ ]

17. In a typical week, on how many days do you do moderate-intensity physical activities (like brisk walking, climbing the stairs, gardening, doing household chores, etc.) for at least 30 minutes per day? (a) < 5 days [ ] (b) ≥ 5 days [ ]

18. In a typical week, on how many days do you engage in vigorous intensity physical activities (like running, carrying or lifting heavy loads, bicycling, football playing, tennis playing, etc.) for at least 20 minutes per day? (a) < 3 days [ ] (b) ≥ 3 days [ ]

19. On most days, how many hours do you sleep in the night? (a) < 7 hours [ ] (b) ≥ 7 hours [ ] (c) I don’t know [ ]

SECTION D: PERCEPTION OF LIFESTYLE AND COVID-19

Kindly indicate to what extent to which you agree with the statements below, stating whether you – Strongly Agree (SA), Agree (A), Indifferent (I), Disagree (D), or Strongly Disagree (SD).

20. Regular consumption of fruits and vegetables can protect people from contracting the COVID-19 virus.

21. Regular consumption of fried and processed foods/drinks like meat pie, donut, cakes, fizzy drinks can increase the chances of people from contracting the COVID-19 virus.

22. Some foods (like soy milk and mushrooms) can prevent people from contracting the COVID-19 virus.

23. Some fish (like tuna, mackerel, herring, and salmon) can prevent people from contracting the COVID-19 virus.

24. Regular exposure to sunlight can reduce people’s chances of contracting the COVID-19 virus.

25. People that are overweight or obese are at higher risk of contracting the COVID-19 virus compared to people with normal weight.

26. People with underlying chronic diseases (like hypertension and diabetes mellitus) are at a higher risk of developing a severe form of COVID-19 compared to people that do not have any of these diseases.

27. People with underlying chronic diseases (like hypertension and diabetes mellitus) are at a higher risk of dying from COVID-19 compared to people that do not have any of these diseases. Thank you
